# Macrophages enhance lipopolysaccharide induced apoptosis via Ang1 and NF-κB pathways in human umbilical vein endothelial cells

**DOI:** 10.1038/s41598-021-82531-7

**Published:** 2021-02-03

**Authors:** Guo-Long Cai, Zhou-Xin Yang, Dong-Yang Guo, Cai-Bao Hu, Mo-Lei Yan, Jing Yan

**Affiliations:** Department of Critical Care Medicine, Zhejiang Hospital, Hangzhou, 310013 Zhejiang China

**Keywords:** Cytokines, Sepsis

## Abstract

Lipopolysaccharide (LPS) could induce apoptosis and dysfunction of endothelial cells. We aimed to reveal the effects of macrophages on cell proliferation and apoptosis in LPS induced human umbilical vein endothelial cells (HUVECs). THP-1 derived macrophages and HUVECs were co-cultured in the presence of LPS. Cell viability was measured by Cell Counting Kit-8 and apoptosis was analyzed by flow cytometry. Expression of Ang1, the NF-κB component p65 was evaluated by western blot and quantitative PCR. Small interfering RNAs (siRNAs) were used to knockdown the expression of proinflammatory cytokines and p65 in HUVECs. Plasmid transfection-mediated overexpression of Ang1 was employed to see its effects on cell proliferation and apoptosis in HUVECs. Macrophages enhanced LPS-induced proliferation impairments and apoptosis in HUVECs, which could be attenuated by siRNA-mediated knockdown of cytokines TNF-α, IL-1β, IL-6 and IL-12p70 in macrophages. The dysfunction of HUVECs was tightly associated with reduced Ang1 expression and increased phosphorylated p65 (p-65). Overexpression of Ang1 in HUVECs significantly decreased p-p65, suggesting negatively regulation of p-p65 by Ang1. Overexpression of Ang1, adding recombinant Ang1 or silencing of p65 substantially attenuated the dysfunction of HUVECs in terms of cell proliferation and apoptosis. In conclusions, THP-1-derived macrophages enhance LPS induced dysfunction of HUVECs via Ang1 and NF-κB pathways, suggesting new therapeutic targets for sepsis.

## Introduction

Sepsis represents a series of common diseases in intensive care unit (ICU), which causes many deaths worldwide. In sepsis, the dysfunction of endothelial cells is considered one of the central events, as they potently decrease vascular permeability, cause endothelial barrier damage and further organ dysfunction. Lipopolysaccharide (LPS) is a component of gram-negative bacteria, and it can be recognized by toll-like receptor 4, which express many kinds of cells including endothelial cells. LPS could induce apoptosis and dysfunction of endothelial cells^1,2^. Meanwhile, LPS actives many kinds of immune cells to induce the secreting of proinflammatory cytokines from these cells. Moreover, many proinflammatory cytokines also cause dysfunction of endothelial cells^3^.

Accumulating studies have well documented that macrophages play an important role in sepsis as it is one of the major sources of tumor necrosis factor-a (TNF-a) and interleukin-6 (IL-6). Macrophages can be derived from monocytes. For example, monocytes within the bloodstream adhere to endothelia cells and differentiate into macrophages. Since macrophage is capable of mediating cytotoxic effects and is of great importance in aggressive inflammatory response in sepsis, we speculated that macrophages play roles in the dysfunction of endothelial cells during sepsis.

As an essential regulator of vascular development, Angiopoietin-1 (Ang1) functionally regulates many aspects of endothelial cells. It has been demonstrated that angiogenesis, survival, cell migration and tube formation of endothelial cells are promoted by Ang1^4,5^. Ang1 binds to its receptor tyrosine kinase Tie2, and protects endothelial cells from apoptosis and LPS-induced endotoxic shock^6^. Signal transduction pathways like PI3K/Akt pathway, NF-κB pathway and p38 MAPK pathway have been shown to be important for Ang1 mediated cellular effects^7,8^. Moreover, high concentrations of Ang2 (a competitive antagonist for Ang1) in plasma are associated with organ dysfunction and mortality in human septic shock^9^. Thus, Ang1 is critical for sepsis and its regulation is of great importance for sepsis control. Besides, NF-κB pathway has been suggested to be critical for classical TLR signaling and sepsis. Activation of NF-κB pathway causes transcription of proinflammatory cytokines like TNF-α and IL-6 in macrophages^10^. However, the roles of Ang1/2 and NF-κB pathway in macrophages induced dysfunction of endothelial cells have not been clearly elucidated yet.

THP-1 is a human monocytic cell line derived from an acute monocytic leukemia patient, and these cells have the potential to differentiate into macrophages, which makes the THP-1 cell line as a valuable tool for investigating the functions of human macrophages in both health and disease. In this study, we co-cultured THP-1-derived macrophages with human umbilical vein endothelial cells (HUVECs), and LPS was added to investigate the alterations on cell proliferation and apoptosis of HUVECs. We found that addition of macrophages in the culture significantly enhanced LPS induced proliferation impairment and apoptosis of HUVECs, through soluble factors like TNF- α, IL-1β, IL-6 and IL-12p70. Importantly, Ang1 negatively regulated the phosphorylation of p65 in HUVECs. Our study suggested that Ang1/2 and NF-κB pathway play a central role in regulating macrophages mediated and LPS induced dysfunction of HUVECs.

## Materials and methods

### Cell culture

The THP-1 cell line was purchased from the American Type Culture Collection, and were grown in RPMI-1640 media containing 10% FBS (Hyclone) and antibiotics (100 U/mL penicillin and streptomycin). HUVECs were purchased from the cell bank of Chinese Academy of Sciences. HUVECs were cultured in M199 medium (Gibco) including 15% FBS, antibiotics, 30 μg/mL EGFS(Sigma) and 10 ng/mL epidermal growth factor (Sigma). HUVECs of 3th to 6th passages were used in this study.

Medium used for HUVECs single culture were used for the co-culture experiments. 10^5^ HUVECs were seeded in the lower wells of the transwell (6 well plate). THP-1 cells of the same density were plated in the upper chamber of the transwell plate and placed in another 6-well plate. After stimulation by PMA for 48 h, THP-1 cells were cultured for additional 24 h in PMA-free medium to allow full differentiation to single layer macrophages. The upper chamber with macrophage was inserted back to the transwell plate in which HUVECs were growing in the bottom wells. 100 μg/mL LPS (Sigma) were used for treatment of the cells. All experimental procedures used had been approved by the ethics committee of Zhejiang Hospital.

### Cell proliferation assay and apoptosis analysis

Cell Counting Kit-8 (Dojindo Molecular Technologies) were used for measurement of the cell viability of HUVECs under various treatments. Apoptosis of HUVECs were analyzed by Apoptosis Detection Kit (BestBio). HUVECs were isolated and resuspended in 100 μL PBS. Annexin V (5 μL) and propidium iodide (10 μL) were add and incubated in dark for about 20 min. Flow cytometer (FACS Calibur, Becton–Dickinson) were used to detect apoptosis of HUVECs.

### Transfection of siRNA

HUVEC were transfected with siRNA purchased from GenePharma by the Hiperfect Transfection Reagent (Qiagen) as described in Ref.^11^. Briefly, siRNA (100 nM) and transfection reagent (6 μL) were added to serum-free M199 medium, and incubated for 10 min to form transfection complex. Then the transfection complex was added to cultured HUVECs or THP-1 cells. HUVEC or THP-1 cells were incubated with the transfection complexes at 37 °C for 72 h and then the expression of a specific gene was detected by western blot to assess silence efficiency. The targeted sequences for each gene are as follows:

IL­1β siRNA:

GGCCAGGAUAUAACUGACUTT/AGUCAGUUAUAUCCUGGCCTT

TNFα siRNA:

GCGUGGAGCUGAGAGAUAATT/UUAUCUCUCAGCUCCACGCTT

IL­6 siRNA:

CUUCCAAUCUGGAUUCAAUTT/AUUGAAUCCAGAUUGGAAGTT

IL-12p40 siRNA:

CCCUGACCAUCCAAGUCAATT/UUGACUUGGAUGGUCAGGGTT

p65 siRNA:

CCCUAUCCCUUUACGUCAUTT/AUGACGUAAAGGGAUAGGGTT

Negative control:

UUCUCCGAACGUGUCACGUTT/ACGUGACACGUUCGGAGAATT

### Over expression of Ang1 in HUVECs

The cDNA of human Ang1 was purchased from Beneral Biosystems. The open reading frame of Ang1 was subcloned into the mammal expression vector pcDNA3.1 (Atagenix) via EcoRI and HindIII sites by T4 ligation method. HUVEC cells were transfected with the plasmid encoding Ang1 by the Hiperfect Transfection Reagent (Qiagen) according to the manufacturer’s instructions.

### Measurement of proinflammatory cytokines by ELISA

Cell supernatant was collected at indicated time points. The concertation of TNF-α, IL-1β, IL-6 and IL-12p70 were measured by kits from Bio-Swamp via ELISA following the protocols from the manufactures.

### Western blot

Western blot was done as described in Ref.^11^. Briefly, HUVECs or THP-1 cells were lysed in Radio immunoprecipitation assay buffer with protease inhibitor cocktail (Roche). After measuring the protein concentration, 50 μg protein was used to electrophoresed in sodium dodecyl sulfate–polyacrylamide gel, and transfer it to PDVF membrane (Amersham Biosciences). The transferred membrane was sealed with 5% BSA at room temperature, and incubated with primary antibody at 4 °C overnight. Then the membrane was washed with Tris-buffered saline-Tween 20 solution, and incubated with secondary antibody at room temperature for 1 h. The band was visualized with imaging system after washing. The following antibodies were used in this study: Ang1 (Proteintech); p65 (AFFINITY); p-p65 (CST); GAPDH (AtaGenix); IL-1β (Proteintech); TNF-α (Proteintech); IL-6 (Proteintech); IL12p40 (abcam).

### Statistical analysis

Data were analyzed for statistical significance using the GraphPad Prism software. Data are expressed as mean ± SD. Unpaired student's *t* test or ANOVA (Analysis of Variance) were used to determine the significance between two groups or multiple groups, and p < 0.05 was considered to be statistically significant.

### Ethical approval

All experimental procedures used in this study had been approved by the ethics committee of Zhejiang Hospital.

## Results

### THP-1-derived macrophages enhance LPS-induced proliferation impairment and apoptosis of HUVECs

**Figure 1:**
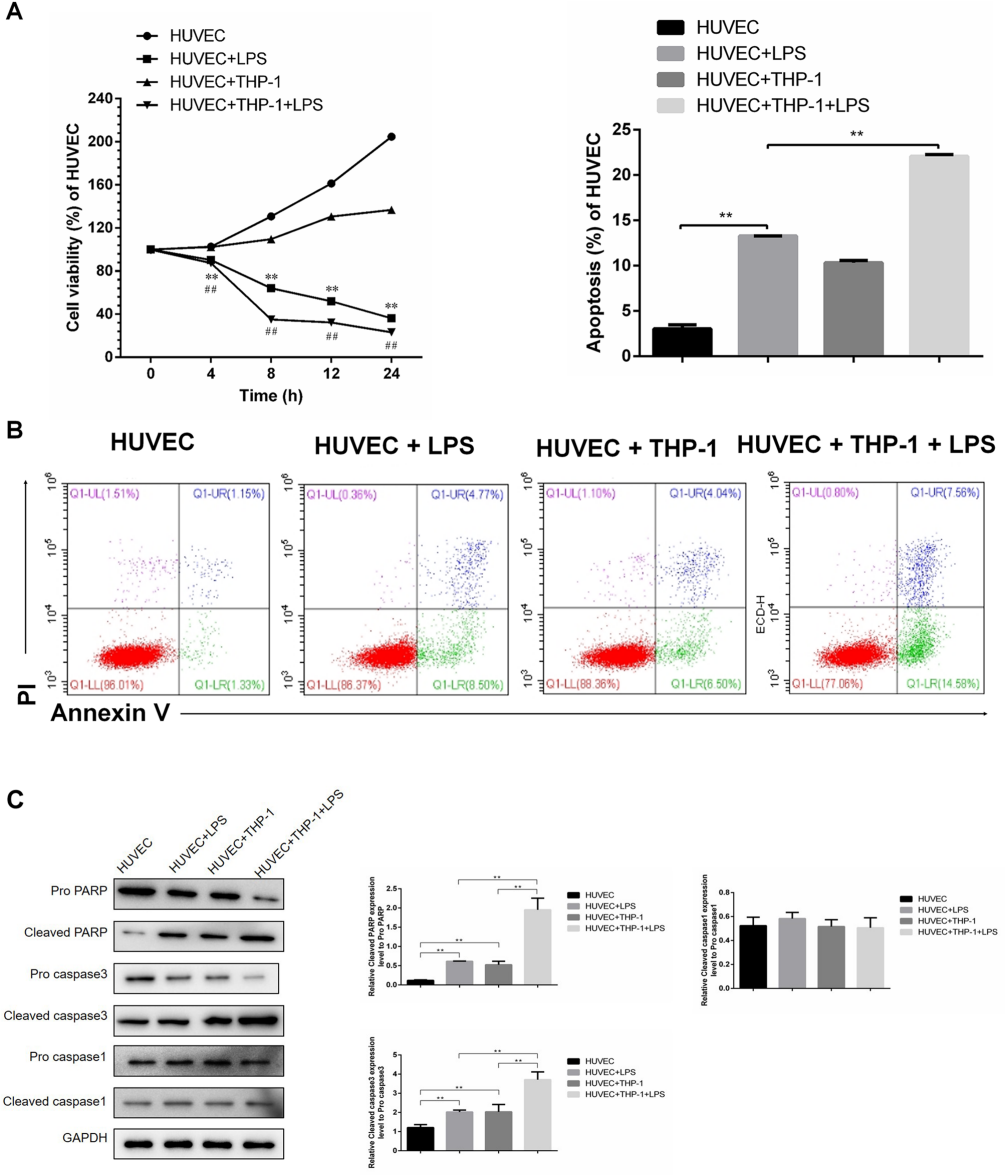
THP-1-derived macrophages enhanced LPS-induced proliferation impairment and apoptosis of HUVECs. Four groups of HUVECs, including control HUVEC without treatment or co-culturing (HUVEC), HUVEC treated with 100 μg/mL LPS alone (HUVEC + LPS), HUVEC co-cultured with THP-1-derived macrophages (HUVEC + THP-1) and LPS stimulated HUVECs that co-cultured with THP-1-derived macrophages (HUVEC + THP-1 + LPS), were measured for cell proliferation by CCK-8 assay (**A**) and apoptosis by flow cytometry (**B**) after 8 h culture. Apoptosis of HUVECs was quantitated by Annexin V and PI staining via flow cytometry. Annexin V^+^ cells are considered as apoptotic cells. n = 3 for each group. Data are expressed as mean ± SD.

Our investigation focused on whether and how macrophages affect the functions of HUVECs. Previous studies have established the roles of LPS on apoptosis and dysfunction of endothelial cells. Therefore, in our experiment designs, we set up four groups, including control HUVEC without treatment or co-culturing (HUVEC), HUVEC treated with LPS alone (HUVEC + LPS), HUVEC co-cultured with THP-1-derived macrophages (HUVEC + THP-1) and LPS stimulated HUVECs that co-cultured with THP-1-derived macrophages (HUVEC + THP-1 + LPS), to compare their cellular proliferation and apoptosis. While co-culturing with THP-1 cells slightly suppressed proliferation of HUVECs, LPS stimulation significantly inhibited HUVEC proliferation, as indicated by around 50% reduction of cell viability starting from 8 h. Adding THP-1 macrophages to the culture would further down-regulate the cell viability, as evidenced by over 50% decrease of the cell proliferation at 8 h after co-culturing (Fig. 1A). Similar trends regarding apoptosis were observed when HUVECs under various conditions were stained by Annexin V and PI. The stimulation of LPS substantially increased the rate of apoptotic cells (around 12%, compared to base rate of ~ 3% for control HUVECs). This effect was further enhanced when THP-1-derived macrophages were added in the culture, which resulted in around 20% of apoptotic rate (Fig. 1B). PARP (poly ADP-ribose polymerase) and caspase-3 have been shown to be critical in apoptosis. Therefore, we analyzed the expression of PARP and caspase-3. While either LPS stimulation alone or co-culturing with THP-1 cell alone significantly increase the expression of cleaved PARP and cleaved caspase-3 in HUVECs, administration of both (LPS stimulation plus macrophage co-culturing) synergistically further reduced the expression of cleaved PARP and cleaved caspase-3 protein in HUVECs (Fig. 1C). The expression of cleaved caspase-1 maintained after LPS treatment and co-culturing with THP-1 cell (Fig. 1C), suggesting LPS did not induce HUVEC pyroptosis in our study. These results suggested that the dysfunction of HUVEC can be regulated by THP-1-derived macrophages.

### Proinflammatory cytokines enhanced LPS-induced proliferation impairment and apoptosis of HUVECs

**Figure 2:**
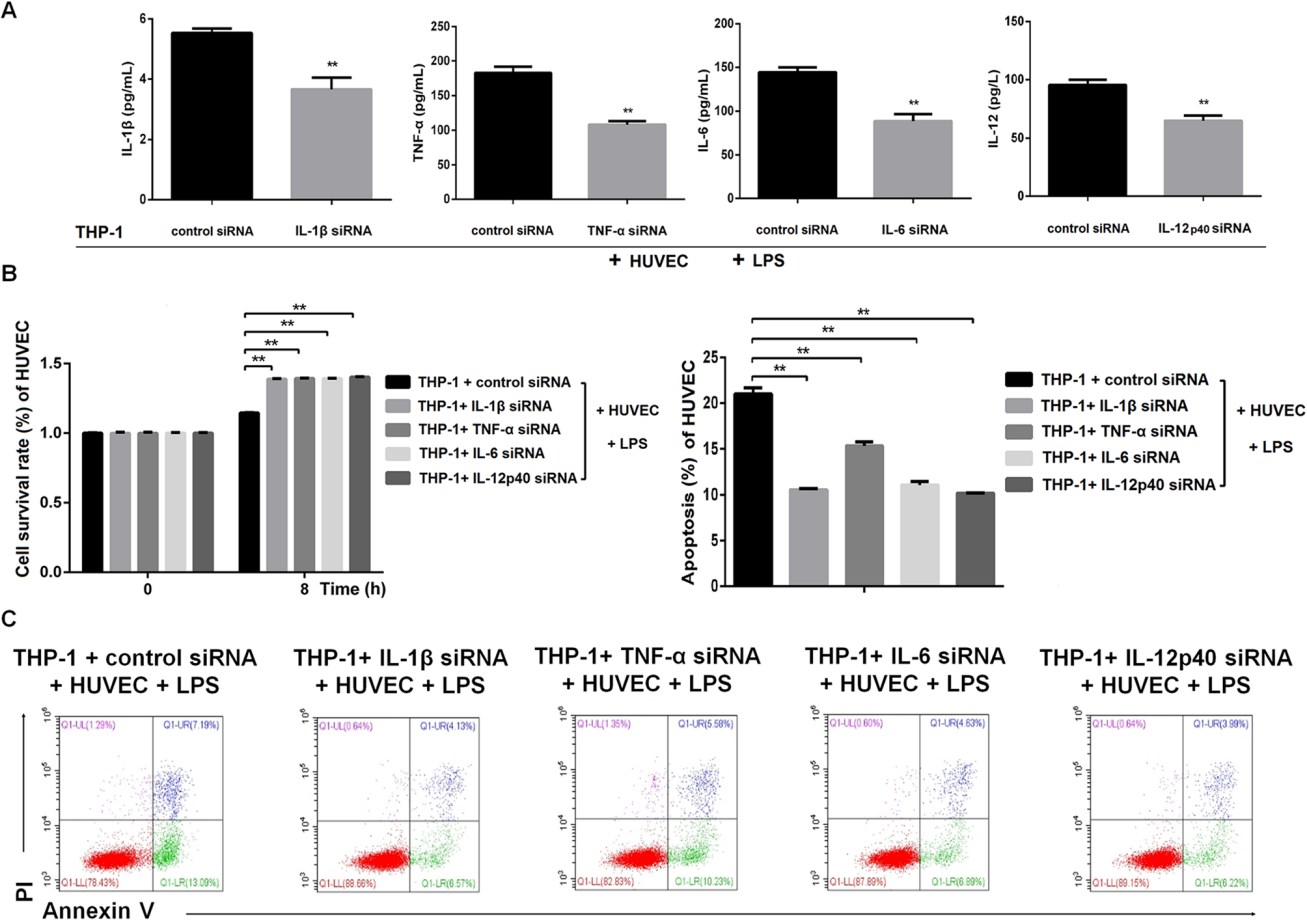
Knockdown of proinflammatory cytokines in THP-1 cells attenuated LPS-induced dysfunction of HUVECs. THP-1-derived macrophages were transfected with siRNA oligos targeting IL­1β, TNF­α, IL­6 and IL­12 p40 or control siRNA for 24 h, and then co-cultured with HUVECs upon LPS (100 μg/mL) stimulation for 8 h. (**A**) siRNA-mediated knockdown of IL-1β, TNF-a, IL-6 and IL-12p40 in THP-1-derived macrophages resulted in significant reduction of soluble cytokine levels in the supernatant of the co-culturing, as measured by ELISA. (**B**) siRNA-mediated knockdown of the proinflammatory cytokines in THP-1-derived macrophages significantly attenuated the impairment of cell proliferation in HUVECS after the co-culture. Cell viability was detected by CCK8 at 0 h and 8 h. (**C**) siRNA-mediated knockdown of the proinflammatory cytokines significantly attenuated the apoptosis of HUVECs after the co-culture. Apoptosis of HUVECs was quantitated by Annexin V and PI staining via flow cytometry. Annexin V^+^ cells are considered as apoptotic cells. n = 3 for each group. Data are expressed as mean ± SD.

LPS induces the production of proinflammatory cytokines (including IL­1β, TNF­α, IL­6 and IL­12) from macrophages. Add THP-1-derived macrophages to HUVEC increased the concertation of TNF-α, IL-1β, IL-6 and IL-12p70. Besides, HUVEC + THP-1 + LPS group also had a higher level of TNF-α, IL-1β, IL-6 and IL-12p70 than HUVEC + LPS group. (Supplementary Fig. 1). We wondered whether the soluble cytokines played a role in suppressing cell proliferation and promoting apoptosis in our HUVEC-macrophage co-culturing experiments. To study the effects of these cytokines, we used siRNA duplexes to knockdown their expressions in THP-1-derived macrophages (Supplementary Fig. 2). First, we validated that the administration of siRNA efficiently down-regulated the cytokine levels in the supernatant of HUVECs co-cultured with siRNA treated THP-1-derived macrophages (Fig. 2A). Knocking down of either cytokine coded gene in THP-1 cells significantly enhanced cell proliferation of HUVECs (Fig. 2B). Accordingly, the silence of either of these genes profoundly reduced apoptosis of HUVECs (Fig. 2C). These results suggest that the cytokines IL­1β, TNF­α, IL­6 and IL­12 were important in regulating cell viability of HUVECs that were co-cultured with LPS treated THP-1-derived macrophages.

### THP-1-derived macrophages regulate Ang1 and NF-κB pathways in HUVECs through LPS-mediated production of proinflammatory cytokines

**Figure 3:**
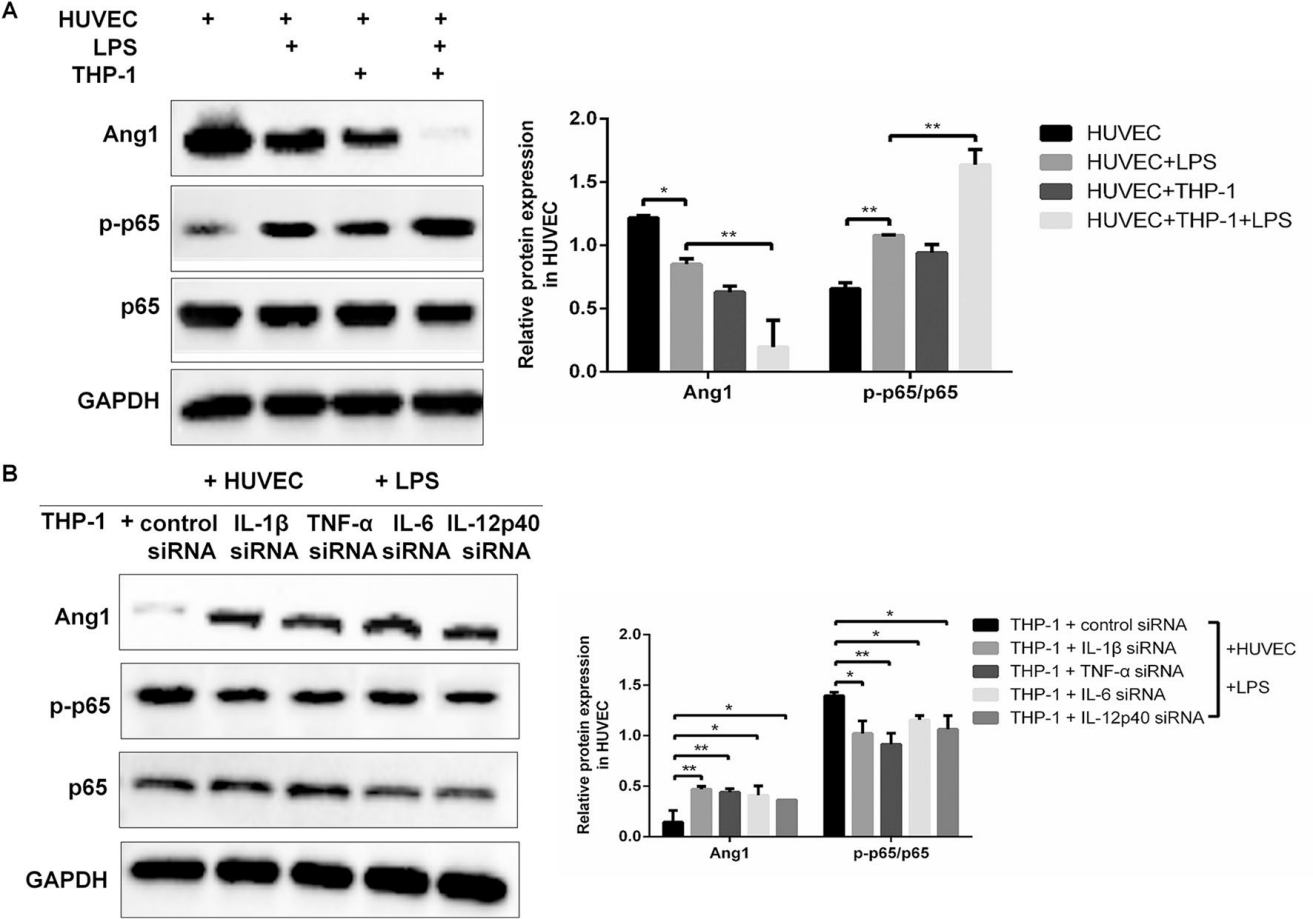
THP-1-derived macrophages regulate Ang1 and NF-κB pathways in HUVECs. (**A**) Protein expressions of Ang1, p65 and p-p65 in HUVECs under four indicated conditions (as set in Fig. 1) were detected by western blot. (**B**) THP-1-derived macrophages were transfected with siRNA targeting IL­1β, TNF­α, IL­6 and IL­12 p40 or control siRNA for 24 h, and then co-cultured with HUVEC in the presence of LPS (100 μg/mL) for 8 h. Protein expressions of Ang1, p65 and p-p65 in the HUVECs were detected by western blot. Relative expression of Ang1 (normalized to GAPDH) and the ratio of p-p65 to p65 were quantified. n = 3 for each group. Data are expressed as mean ± SD.

The Ang1 and NF-κB pathways have been shown to be critical for regulating the function of HUVECs. Therefore, we tested the impacts of LPS stimulation and co-culturing with THP-1-derived macrophages on the expression of Ang1 and p65, an important component of the NF-κB complexes. While either LPS stimulation alone or co-culturing with THP-1 cell alone significantly reduced the expression of Ang1 in HUVECs, administration of both (LPS stimulation plus macrophage co-culturing) synergistically further reduced the expression of Ang1 protein in HUVECs (Fig. 3A). On the contrary, a substantial increase of phosphorylated p65 (p-p65) but not unmodified p65 was observed by either LPS treatment or macrophages co-culturing. Likewise, a further increase of p-p65 (over twofold) in LPS-stimulated and macrophages-co-cultured HUVECs reflected a synergistic effect on the NF-κB pathway (Fig. 3A). The p-p65 can be up-regulated by LPS alone or co-culture with THP-1-derived macrophages, suggesting possible roles of proinflammatory cytokines on regulating Ang1 and p-p65 levels in HUVECs. Indeed, knockdown of either IL­1β, TNF­α, IL­6 or IL­12 p40 in THP-1 cells elevated the expression of Ang1 and significantly reduced the phosphorylation of p65 in co-cultured HUVECs (Fig. 3B). These results suggested that the Ang1 and NF-κB pathways were regulated by proinflammatory cytokines in HUVECs.

### Ang1 negatively regulated the phosphorylation of p65 in HUVECS

**Figure 4:**
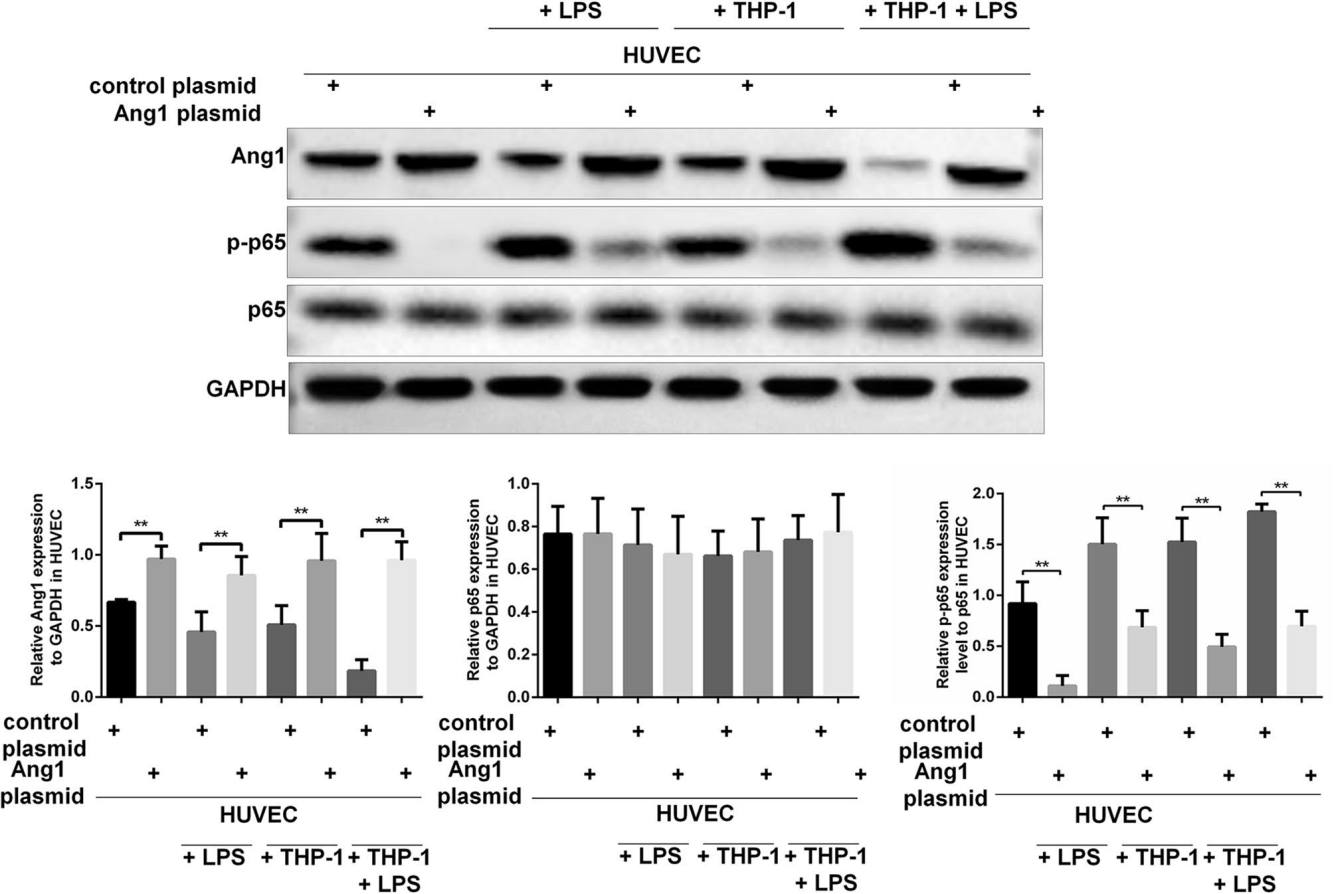
Ectopic expression of Ang1 remarkably reduced the phosphorylation of p65 in HUVECs under various conditions. HUVECs were transfected with Ang1-expressing plasmid or control plasmid for 24 h; and then the HUVECs were cultured alone with/without LPS (100 μg/mL) for 8 h, or co-cultured with THP-1-derived macrophages with/without LPS (100 μg/mL) for 8 h. Protein expressions of Ang1, p65 and p-p65 in the HUVECs were measured by western blot. Relative expressions of Ang1 and p65 (normalized to GAPDH), and the ratio of p-p65 to p65 were quantified. n = 3 for each group. Data are expressed as mean ± SD.

The dysfunction of HUVECs under LPS or macrophages co-culturing mediated stresses was tightly associated with reduced Ang1 expression and increased phosphorylation of p65. We set to examine possible interaction between the Ang1 and NF-κB pathways. Ang1 was over-expressed in HUVECs by plasmids transfection, and the expression levels of p65 and p-p65 were evaluated in HUVECs under various treatments. Plasmid transfection mediated expression of Ang1 was successful, as evidenced by elevated Ang1 protein levels in control HUVECs, LPS stimulated HUVECs, HUVECs co-cultured with THP-1-derived macrophages and LPS-stimulated/macrophages co-cultured HUVECs (Fig. 4). Whereas there were no significant changes regarding the expression levels of p65 (Fig. 4), the levels of phosphorylated p65 (p-p65) were remarkably reduced by forced expression of Ang1 in either group (Fig. 4). These data clearly indicate that Ang1 negatively regulates the phosphorylation of p65 in HUVECs.

### Ang1 and NF-κB pathways play a central role in regulating cell proliferation and apoptosis in HUVECs

**Figure 5:**
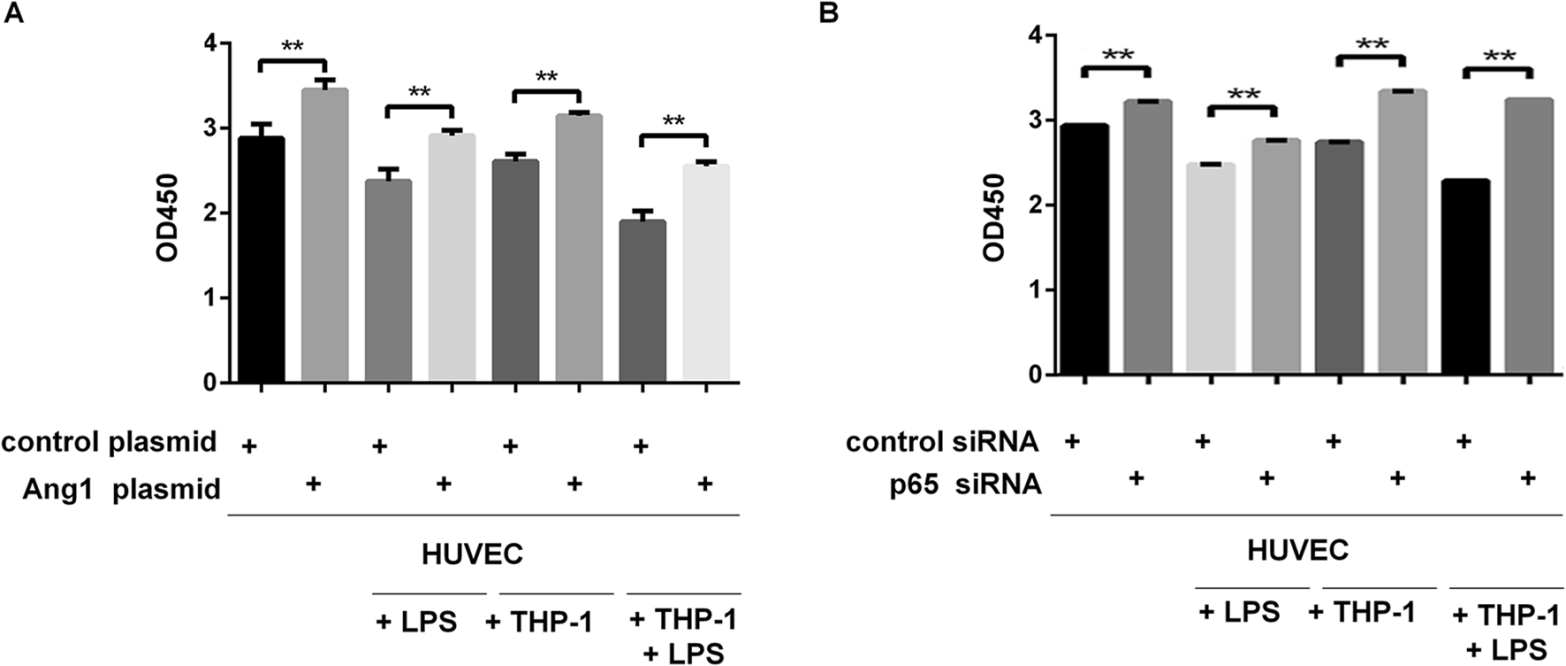
Either forced expression of Ang1 or knockdown of p65 significantly attenuated the impaired cell viability of HUVECs under various conditions. (**A**) HUVECs were transfected with Ang1-expressing plasmid or control plasmid for 24 h; and then the HUVECs were cultured alone with/without LPS (100 μg/mL) for 8 h, or co-cultured with THP-1-derived macrophages with/without LPS (100 μg/mL) for 8 h. Cell viability was assessed by CCK-8 method at 0 h and 8 h after LPS stimulation or co-culturing. (**B**) HUVECs were transfected with p65-targeting siRNA or control siRNA for 24 h; and then the HUVECs were cultured alone with/without LPS (100 μg/mL) for 8 h, or co-cultured with THP-1-derived macrophages with/without LPS (100 μg/mL) for 8 h. Cell viability was assessed by CCK-8 assay at 0 h and 8 h after LPS stimulation or co-culturing. n = 3 for each group. Data are presented as the ratio of 8 h/0 h cell survival rates under indicated conditions, and expressed as mean ± SD.

**Figure 6:**
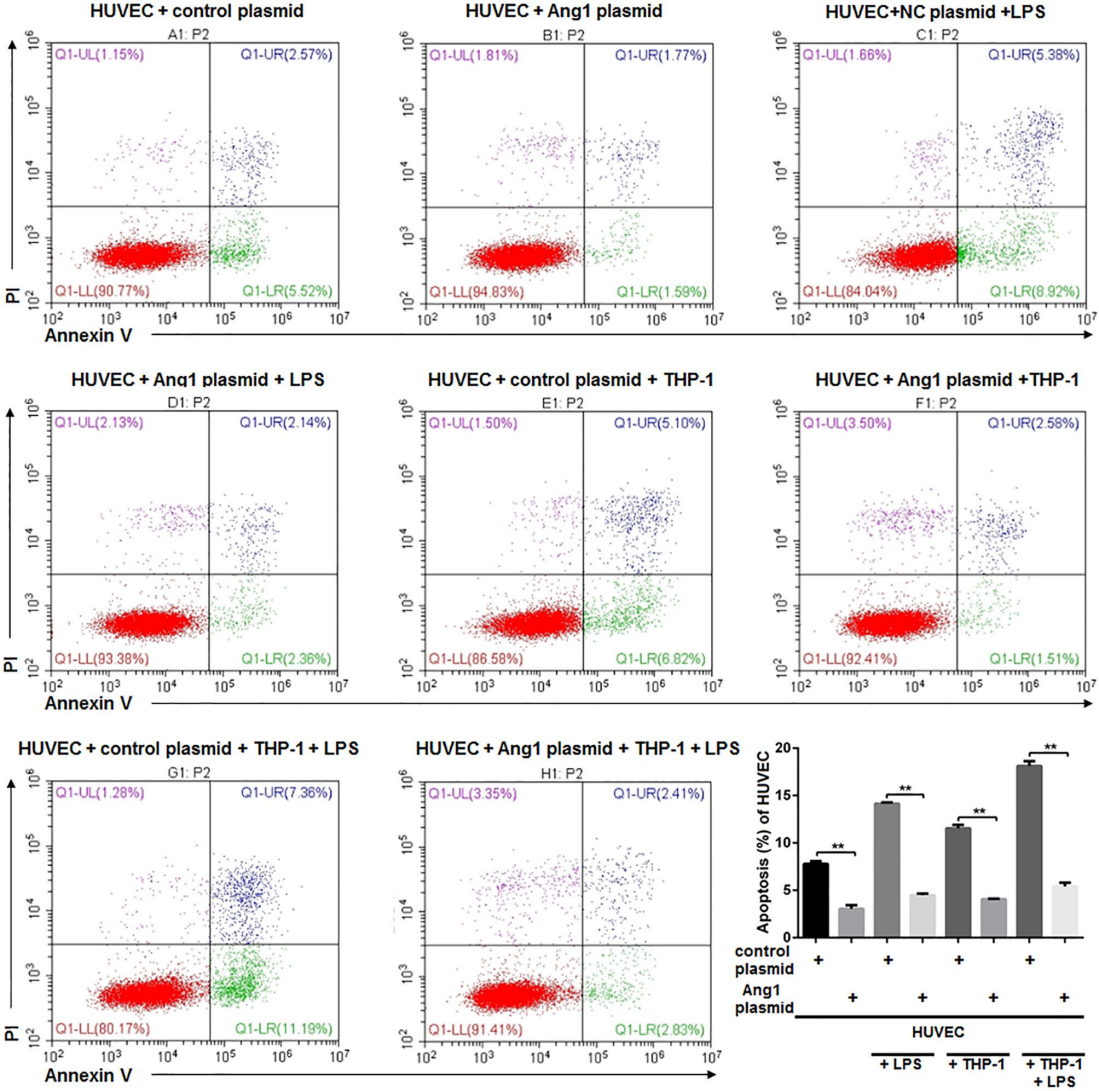
Forced expression of Ang1 significantly attenuated apoptosis of HUVECs under various conditions. HUVECs were transfected with Ang1-expressing plasmid or control plasmid for 24 h; and then the HUVECs were cultured alone with/without LPS (100 μg/mL) for 8 h, or co-cultured with THP-1-derived macrophages with/without LPS (100 μg/mL) for 8 h. Apoptosis of HUVECs was quantitated by Annexin V and PI staining via flow cytometry. Annexin V^+^ cells are considered as apoptotic cells. n = 3 for each group. Data are expressed as mean ± SD.

**Figure 7:**
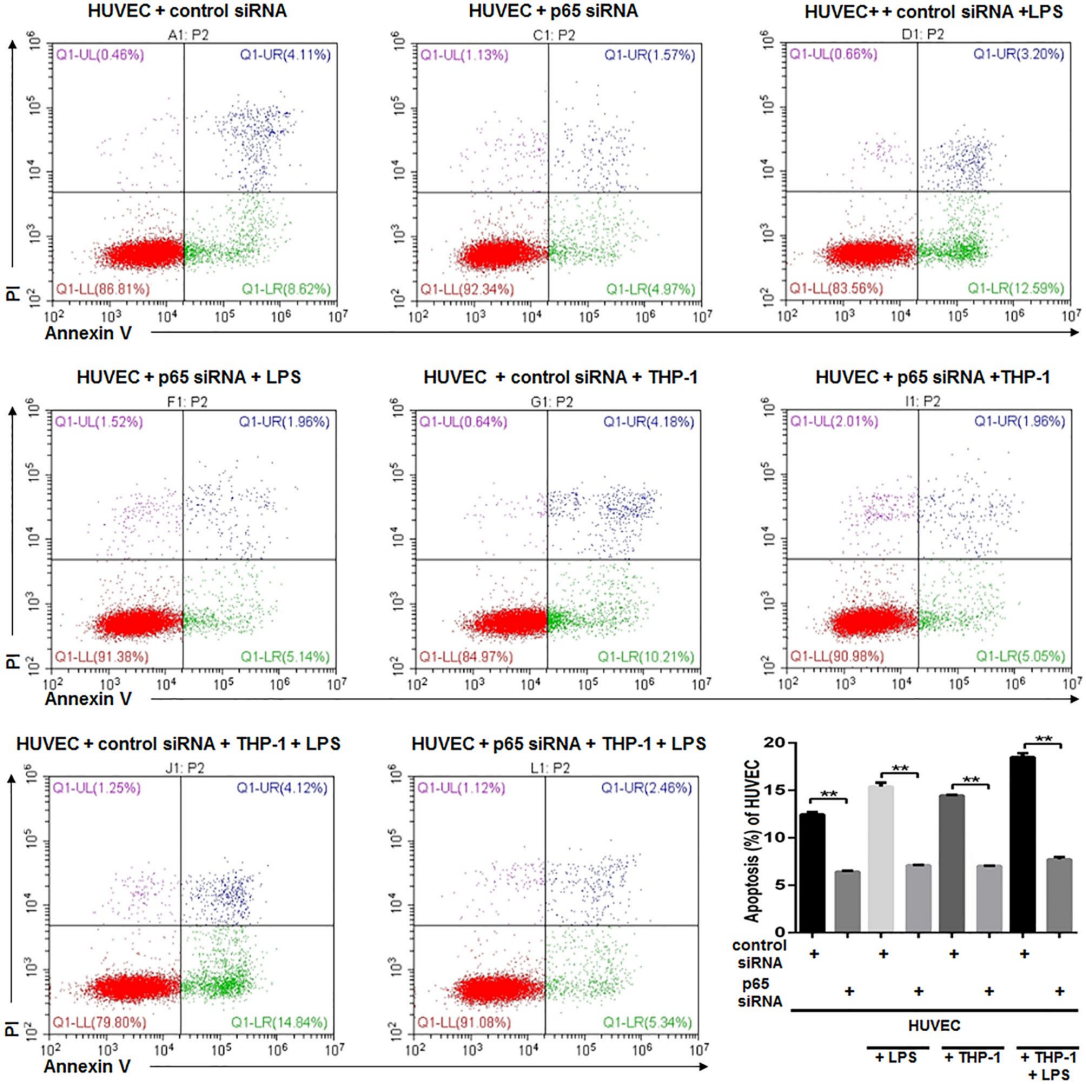
SiRNA-mediated silence of p65 significantly attenuated apoptosis of HUVECs under various conditions. HUVECs were transfected with p65-targeting plasmid or control siRNA for 24 h; and then the HUVECs were cultured alone with/without LPS (100 μg/mL) for 8 h, or co-cultured with THP-1-derived macrophages with/without LPS (100 μg/mL) for 8 h. Apoptosis of HUVECs was quantitated by Annexin V and PI staining via flow cytometry. Annexin V^+^ cells are considered as apoptotic cells. n = 3 for each group. Data are expressed as mean ± SD.

**Figure 8:**
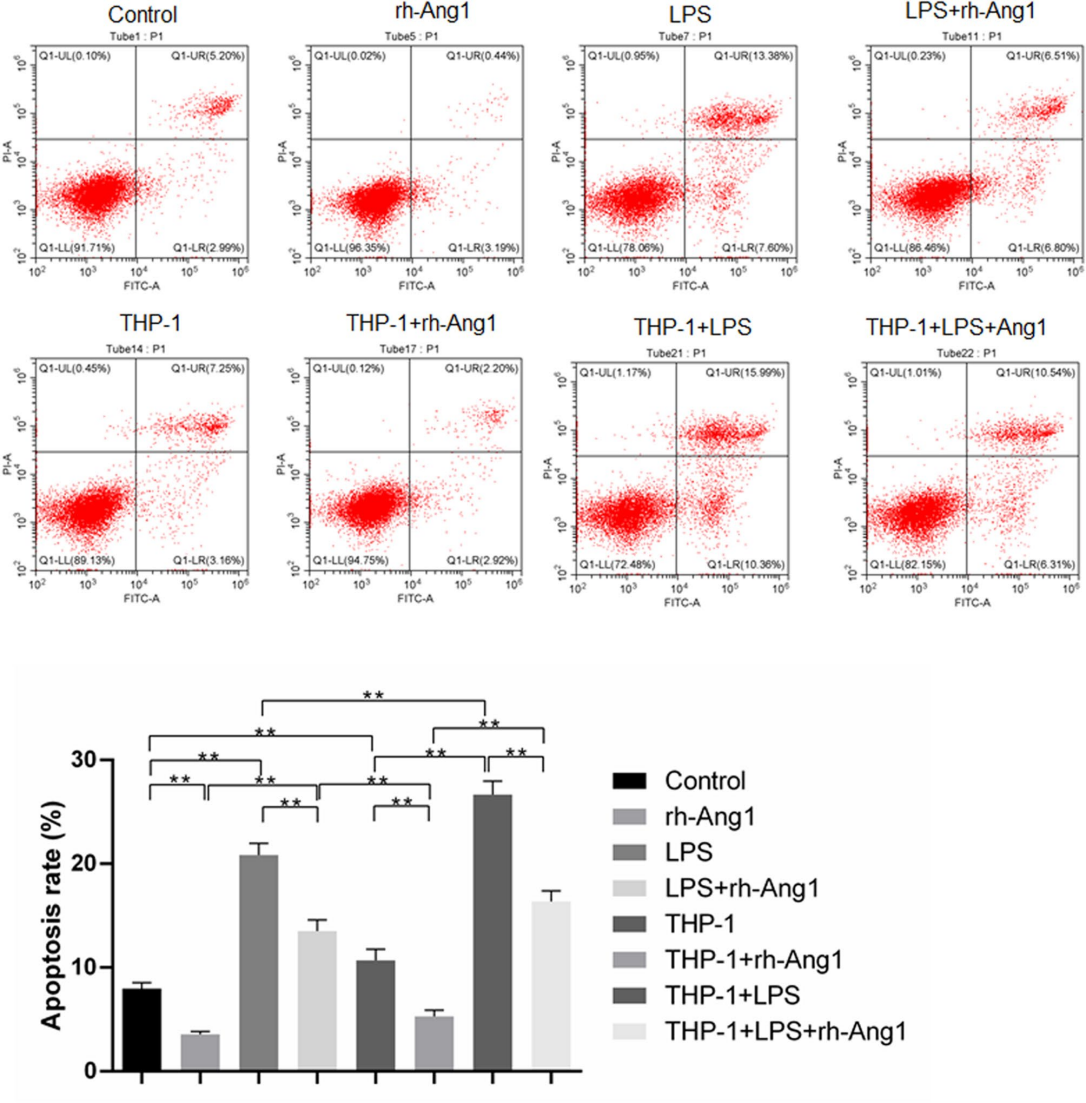
Ang1 significantly attenuated apoptosis of HUVECs under various conditions. Ang1 were used as a final concertation of 250 ng/mL. HUVECs were cultured alone with/without LPS (100 μg/mL) for 8 h, or co-cultured with THP-1-derived macrophages with/without LPS (100 μg/mL) for 8 h. Apoptosis of HUVECs was quantitated by Annexin V and PI staining via flow cytometry. Annexin V^+^ cells are considered as apoptotic cells. n = 3 for each group. Data are expressed as mean ± SD.

The dysfunction of HUVECs in terms of impaired proliferation and increased apoptosis is accompanied by reduced expression of Ang1 and elevated phosphorylation of p65. We used plasmid-mediated Ang1 overexpression and siRNA-mediated knockdown of p65 (Supplementary Fig. 3) to see how Ang1 pathway and NF-κB pathway affected cell viability of HUVECs, respectively. As previous experiments, HUVECs under four different conditions were subjected to proliferation assay at start point (0 h) and 8 h after corresponding treatments. In either of the conditions, elevated expression of Ang1 significantly enhanced proliferation of HUVECs (Fig. 5A). Likewise, the silence of p65 also caused a substantially enhanced cell proliferation, in control HUVECs, LPS-stimulated HUVECs, macrophage co-cultured HUVECs, and HUVECs subjected to both LPS stimulation and macrophage co-culturing (Fig. 5B). Next, we set to investigate the impact of forced expression of Ang1 (Fig. 6) and knockdown of p65 (Fig. 7) on apoptosis of HUVECs. Similarly, the molecular manipulations on either Ang1 or p65 significantly reduced the apoptosis of HUVECs under four tested conditions (Figs. 6, 7). Besides, rhAng1could reduce the apoptosis of the HUVECs under four tested conditions (Fig. 8), further indicating the protection effect of Ang1.

## Discussion

In this study, we demonstrated that THP-1 derived macrophages could enhance LPS-induced proliferation impairment and apoptosis of HUVEC. Proinflammatory cytokines including IL­1β, TNF­α, IL­6 or IL­12 are confirmed to be responsible for promoting the enhanced dysfunction of HUVECs. Importantly, the expression of Ang1 was substantially down regulated by LPS treatment in HUVECs with and without macrophages co-culturing, while more phosphorylation of p65 was observed. Our data suggest an inverse correlation between the expression of Ang1 and the amount for the phosphorylation of p65 in HUVECs. This study highlights the central roles of Ang1 pathway and NF-κB pathway in regulating cell proliferation and apoptosis in HUVECs.

The critical roles of cytokines and NF-κB pathway in mediating dysfunctions of HUVECs were supported by the fact that the proinflammatory cytokines like TNF-α activate NF-κB pathway and cause endothelial dysfunction in patients^12–17^. It have been reported that TNF-α can directly induce apoptosis, while other inflammatory factors are reported less. However, in TNF-α induced HUVEC apoptosis, the concertation of TNF-αwas especially high (20 ng/mL to 200 ng/mL)^18–20^, while TNF-αconcertation in our studies was much lower (lower than 0.5 ng/mL)^21^. Thus, we speculated that inflammatory factors did not induced apoptosis, and they only played a role in promoting LPS-induced HUVEC apoptosis. It was suggested that Ang1 pathway was upstream of NF-κB pathway in endothelial cells, as our data indicate that overexpression of Ang1 down-regulated p-p65, but siRNA-mediated knockdown of p65 had no impact on Ang1 expression. Although Ang1/2 could regulate other pathways like p38 MAPK pathway and PI3K/Akt pathway, NF-κB pathway was critical for endothelial cells apoptosis and dysfunction in our study. However, the regulation of NF-κB by Ang1/2 is not limited in endothelial cells. For example, Ang1-Tie2 could block LPS-induced activation of NF-κB in macrophages^7^. In acute lung injury, Ang1 could block activation of NF-κB, and reduce pro-inflammatory mediators in the serum of ALI rats^22^. As Ang1/2 secreted from endothelial cells regulate many cells, their critical roles on dysfunction of endothelial cells in sepsis still need further clarification.

Macrophages have been suggested to be of great importance for aggressive inflammatory response and immunosuppressive in sepsis^23^. After activation, macrophages could recognize the pathogenic microorganisms, secrete proinflammatory cytokines and activate other immune cells. Proinflammatory cytokines produced by macrophages could affect endothelial cells. Other immune cells also affect endothelial cells. For example, neutrophil could induce myeloperoxidase, causing damage of endothelial cells^24^. Effector T cells cause endothelial cells damage in an Ag-specific manner^25,26^. Nevertheless, our study showed typical way of endothelial cells damage in which macrophages plays critical roles. However, the physiological conditions in patients are much more complex than what we performed for in vitro co-culturing of macrophages alone with HUVECs. Therefore, a sepsis animal model is warranted for the fully elucidation of the roles of macrophages and inflammation in regulating functions of endothelial cells.

The polarization of macrophages could be affected by HUVEC in our study. It have been reported that endothelial cells could produce cytokines like IL-6^27^ and nitric oxide^28^, and these cytokines regulate macrophages^28^. Thus, we speculate that there is a complex interaction between endothelial cells and macrophages. Interestingly, THP-1-derived macrophages without LPS treatment could also affect the viability of endothelial cells. This implies that the recruitment of macrophages to the endothelium may result in risk for endothelium damage. Macrophages constitutively expressed low level of proinflammatory cytokines, which could also regulate the function of endothelial cells. Nevertheless, LPS induced macrophages produced more proinflammatory cytokines and showed stronger effects on endothelial cells.

One of the major limitation of this study is that all of the experiments in this study were done in cultured cells. The process of endothelial injury will be more complicated in vivo, and the role of Ang1/2-NF-κB pathways endothelial injury in remains to be further studied. Nevertheless, our present results have suggested the mechanism of macrophages promoting endothelial injury in vitro cell model, and provided new clues for clinical treatment.

In conclusion, our study suggests that THP-1-derived macrophages enhanced LPS induced apoptosis and dysfunction of HUVECs through Ang1 and NF-κB pathways. These results suggest Ang1/2-NF-κB pathways might be therapeutic targets for sepsis treatment. Further studies with sepsis animal models will provide in depth mechanism of endothelial protection by treatments targeting macrophages and Ang1/2-NF-κB pathways.

## Supplementary Information

Supplementary Information.
