# Supplementary material for: Macrophages enhance lipopolysaccharide induced apoptosis via Ang1 and NF-κB pathways in human umbilical vein endothelial cells
Source: Sci Rep. 2021 Feb 3;11:2918. doi: 10.1038/s41598-021-82531-7 (PMC7858588; doi:10.1038/s41598-021-82531-7)

**Macrophages enhance lipopolysaccharide induced apoptosis via Ang1 and NF-κB pathways in human umbilical vein endothelial cells**

Guo-Long Cai, Zhou-Xin Yang^*^, Dong-Yang Guo, Cai-Bao Hu, Mo-Lei Yan, Jing Yan^*^

Department of Critical Care Medicine, Zhejiang Hospital, Hangzhou, Zhejiang 310013, China

**^*^Correspondence to:**

Zhou-Xin Yang

Department of Critical Care Medicine, Zhejiang Hospital, Hangzhou, Zhejiang 310013, China

Tel: 0086-0571-87377773

E-mail: yangzhouxin@hotmail.com

or

Jing Yan

Department of Critical Care Medicine, Zhejiang Hospital, Hangzhou, Zhejiang 310013, China

Tel: 0086-0571-81595216

E-mail: yanjing201801@163.com

**Running title:** Macrophages enhance HUVECs apoptosis

**Supplementary Figure 1**


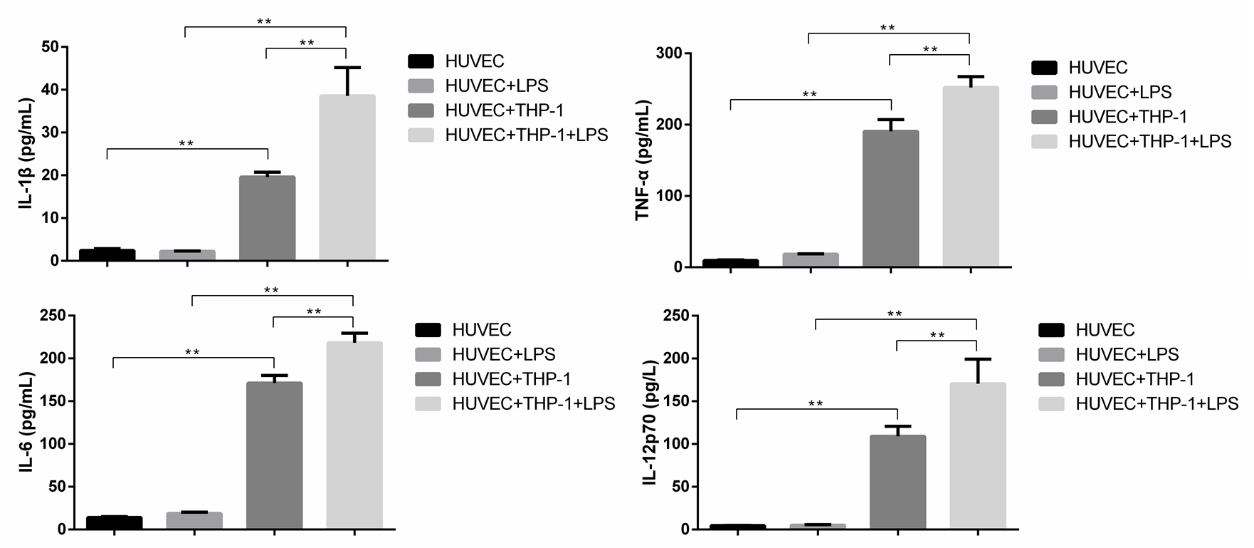


**Supplementary Figure 1. Co-culture of THP-1 cells and HUVEC increase the concertation of proinflammatory cytokines in culture medium.** The culture medium of Four groups of HUVECs (HUVEC, HUVEC+LPS, HUVEC+THP-1 and HUVEC+THP-1+LPS) were isolated, and the concertation of TNF-α, IL-1β, IL-6 or IL-12p70 were tested by ELISA. n=3 for each group. Data are expressed as mean ± SD.

**Supplementary Figure 2**


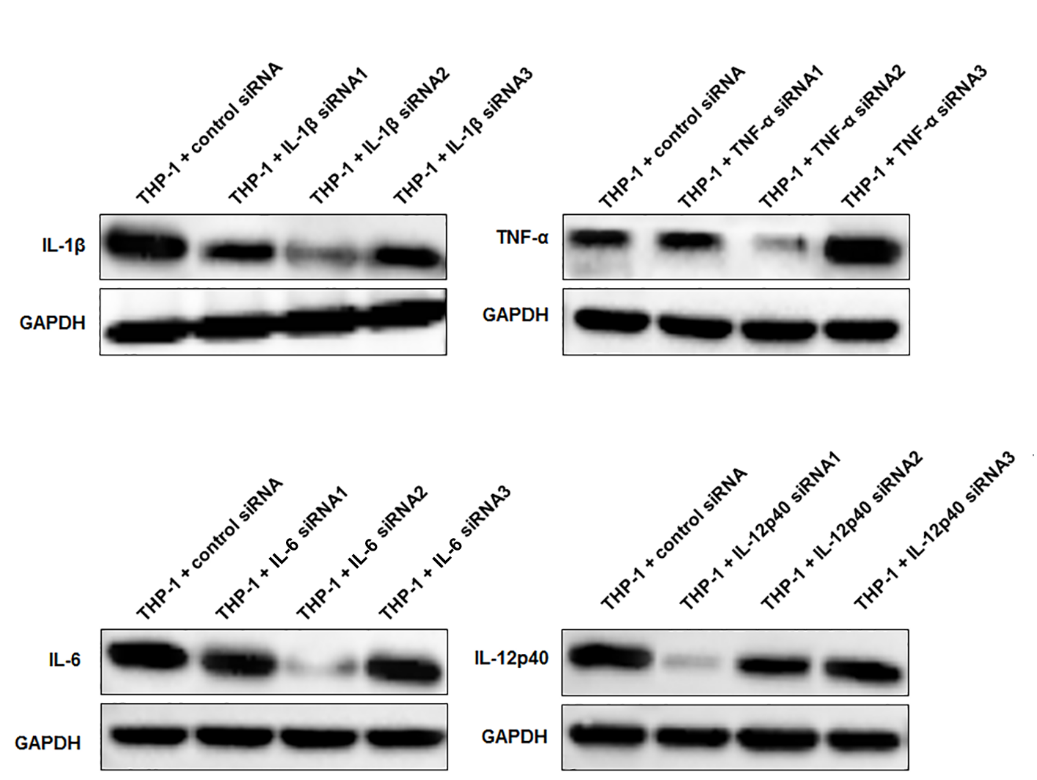


**Supplementary Figure 2. Transfection of designed siRNA oligos markedly reduced the expression of targeted proinflammatory cytokines in THP-1 cells.**

THP-1 cells were transfected with siRNA oligos targeting IL-1β, TNF-1, IL-6 or IL-12p40 for 36 hours, and cells were harvested for western blot analysis to evaluate the knock down effects. Three siRNA oligos for each gene were designed. Image data represent the results from one of three independent experiments. The siRNA oligo with strongest silence effect was selected for subsequent experiments.

**Supplementary Figure 3**

**
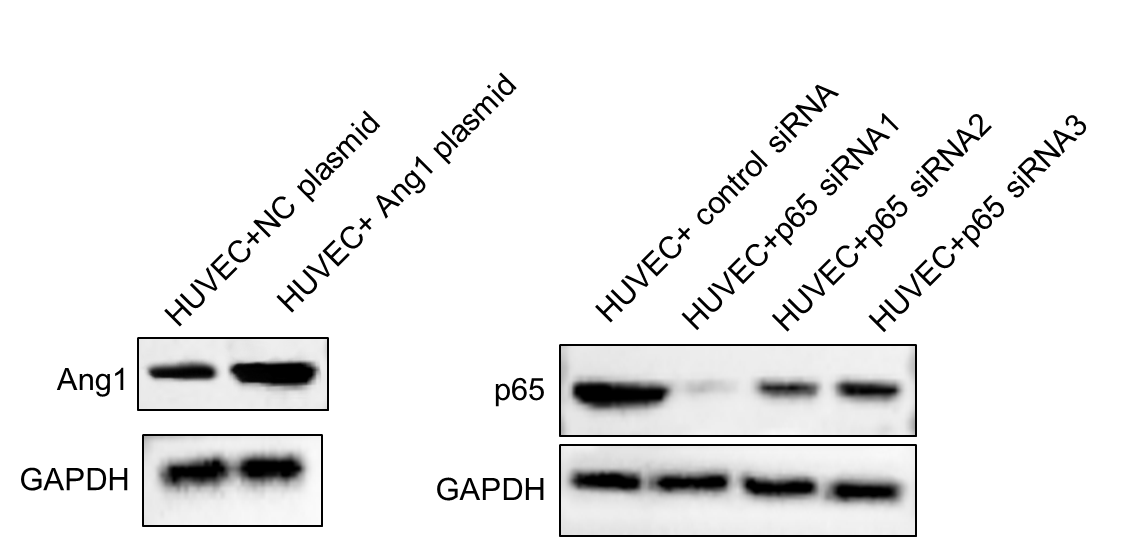
**

**Supplementary Figure 3. Validating the overexpression of Ang1 and knockdown of p65 in HUVECs.**

HUVECs were transfected with Ang1-expressing plasmid or control plasmid for 36 hours, and cells were harvested for western blot analysis to evaluate the overexpression effect. Besides, HUVECs were transfected with p65-targeting siRNA or control siRNA for 36 hours, and cells were harvested for western blot analysis to evaluate the knock down effects. Three siRNA oligos were designed. The siRNA oligo with strongest silence effect was selected for subsequent experiments. n=3 for each group.

**Full gel pictures for Figure 1C**

**pro PARP**


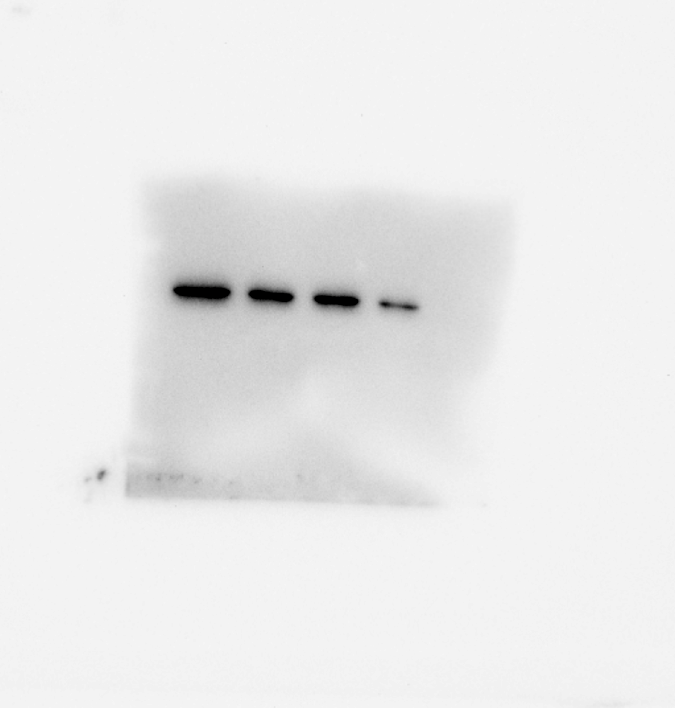


**Cleaved PARP**


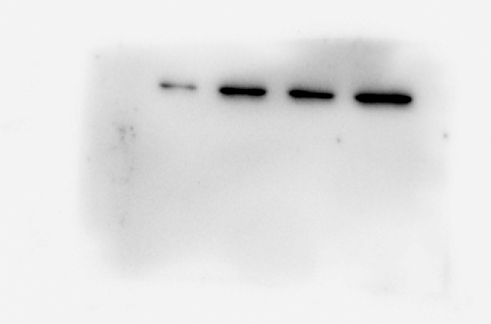


**pro caspase3**


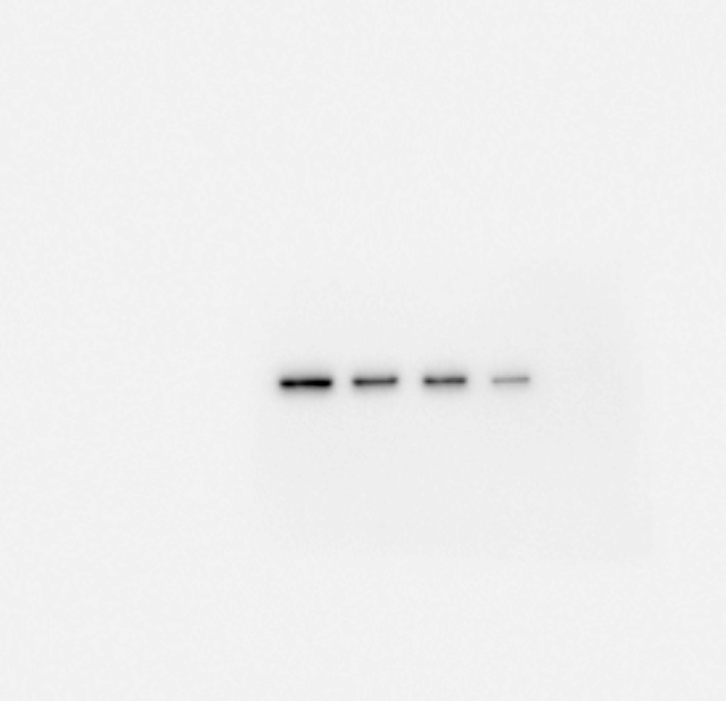


**Cleaved Caspase3**


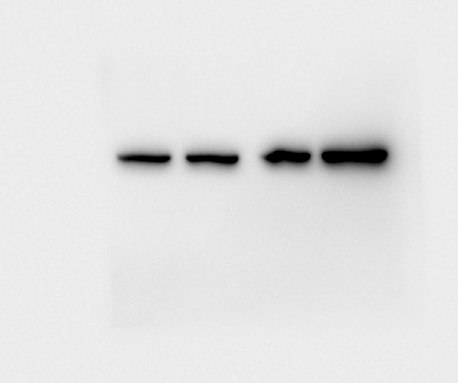


**pro caspase1**


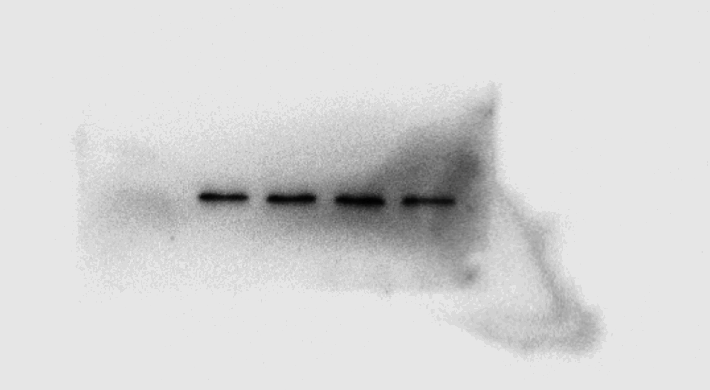


**Cleaved Caspase1**


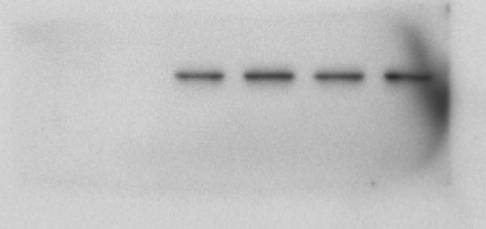


**Full gel pictures for Figure 3A**

**Ang1**


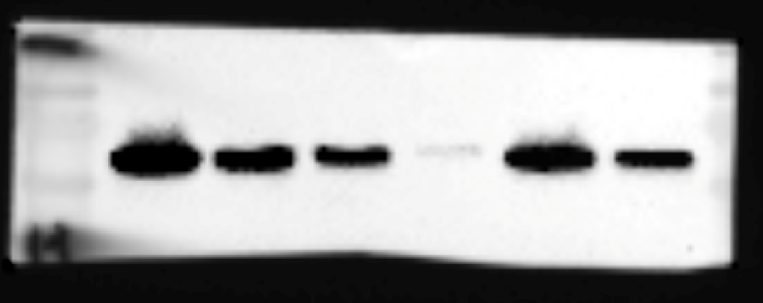


**p-p65**


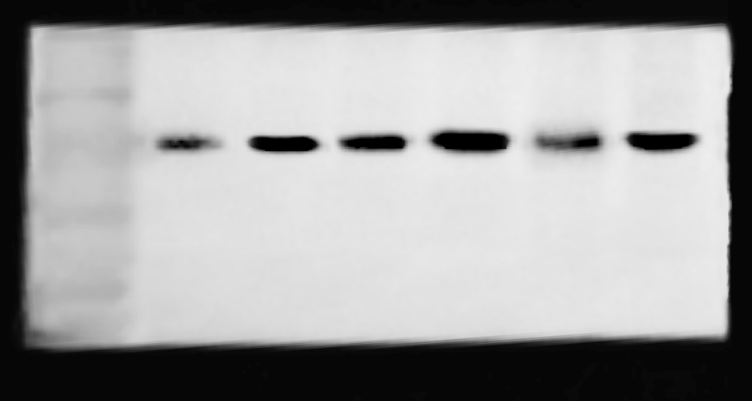


**p65**


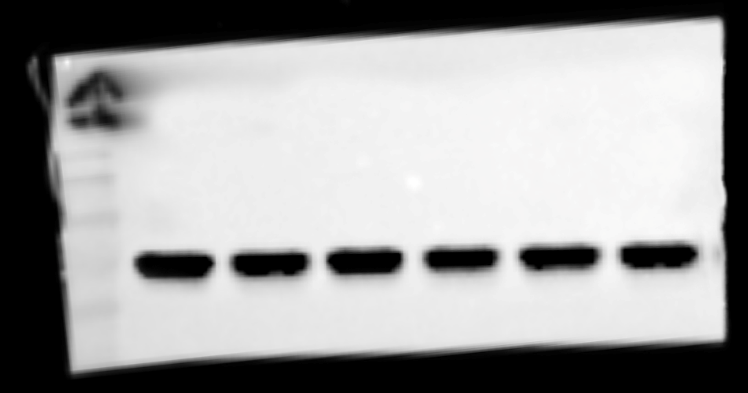


**GAPDH**


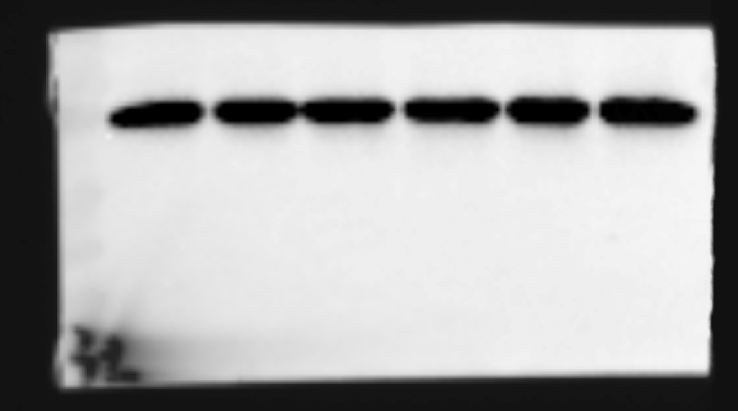


**Full gel pictures for Figure 3B**

**Ang1**


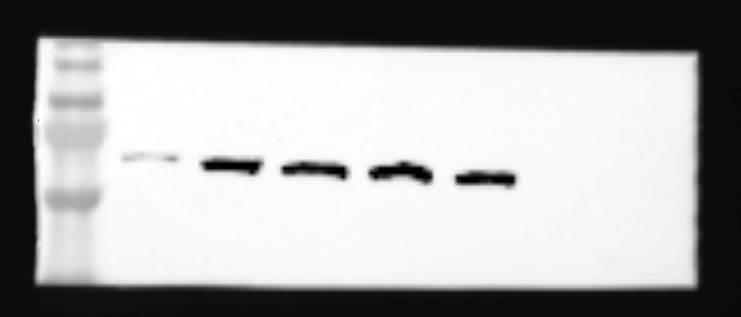


**p-p65**


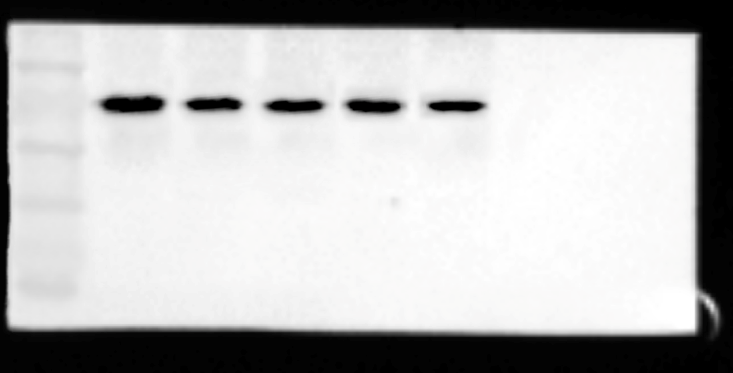


**p65**


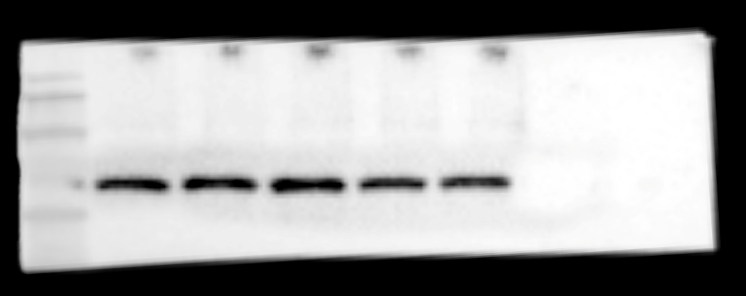


**GAPDH**


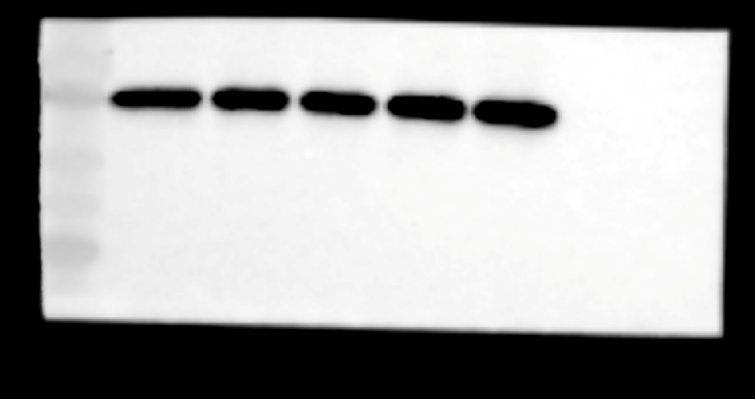


**Full gel pictures for Figure 4**

**Ang1**


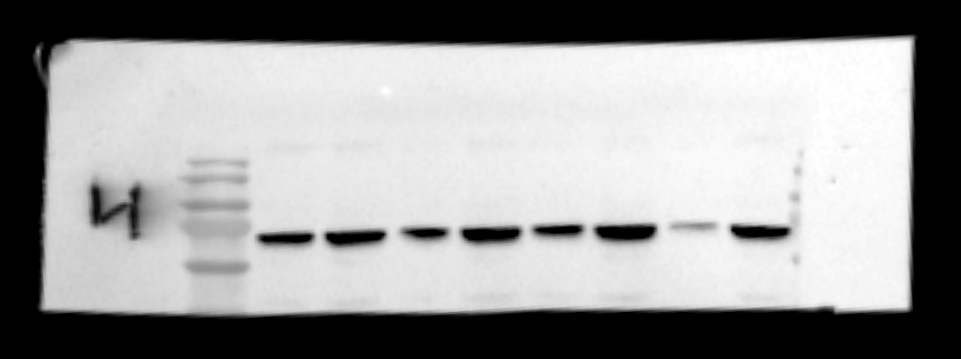


**p-p65**


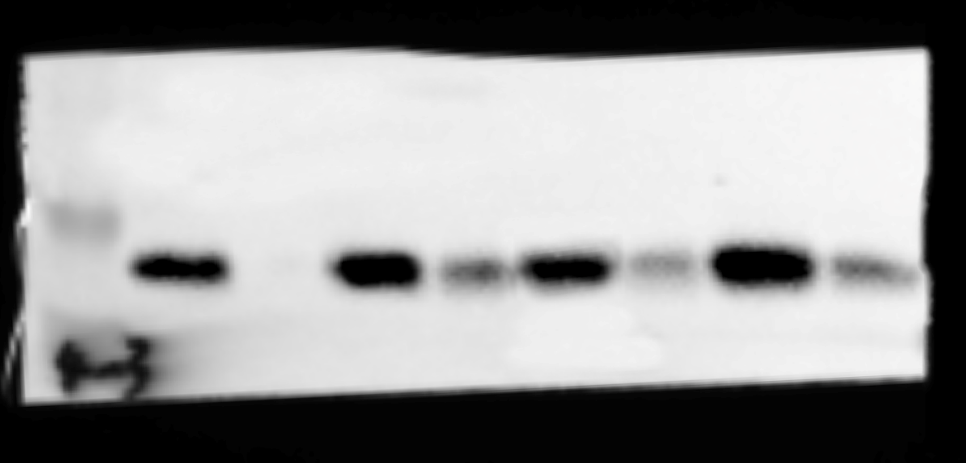


**p65**


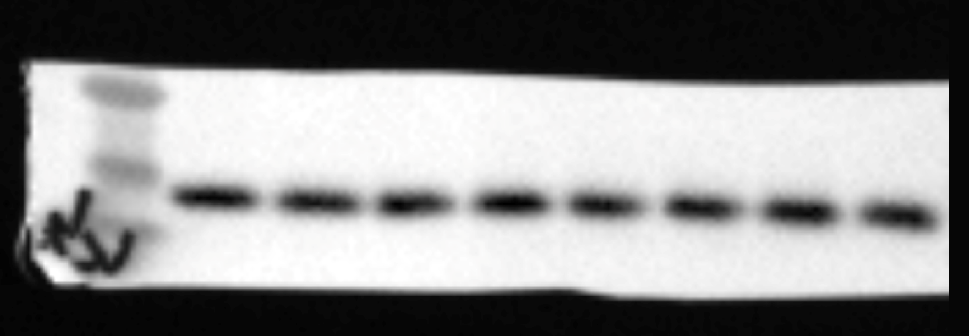


**GAPDH**


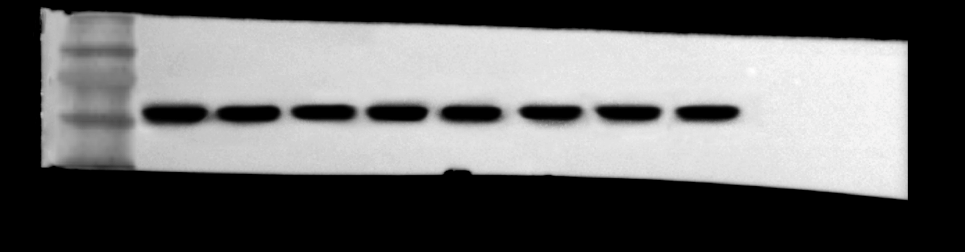


**Full gel pictures for Supplementary Figure 2**

**IL-1β**


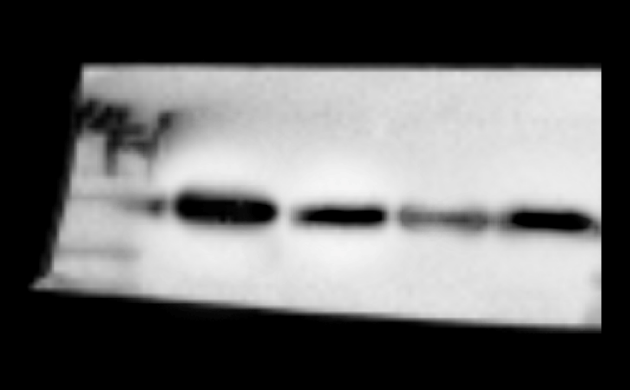


**GAPDH for IL-1β**


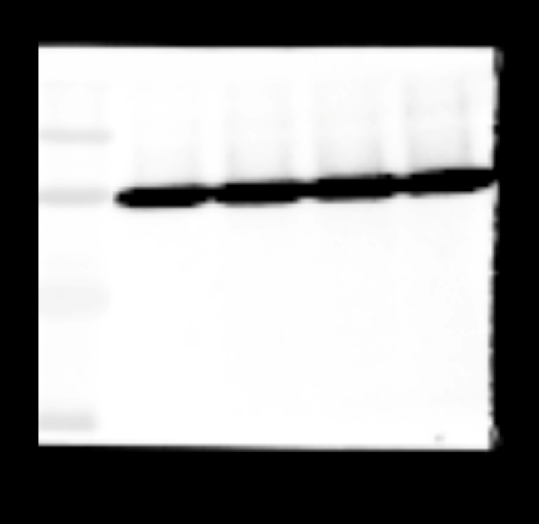


**TNF-α**


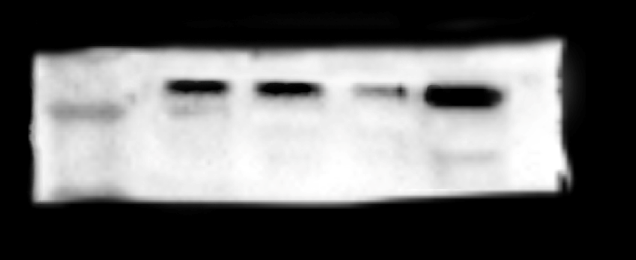


**GAPDH for TNF-α**


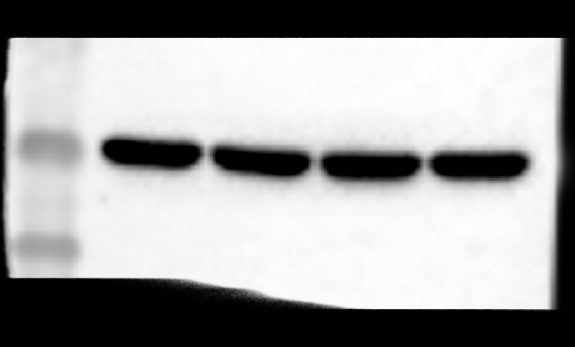


**IL-6**


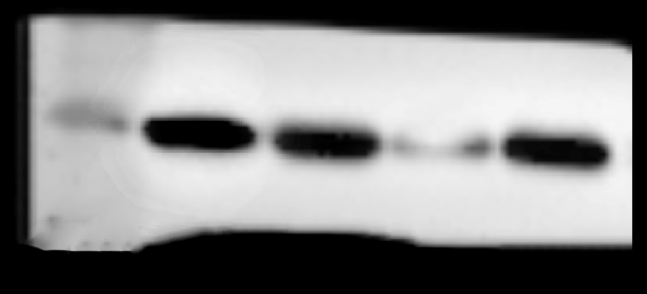


**GAPDH for IL-6**


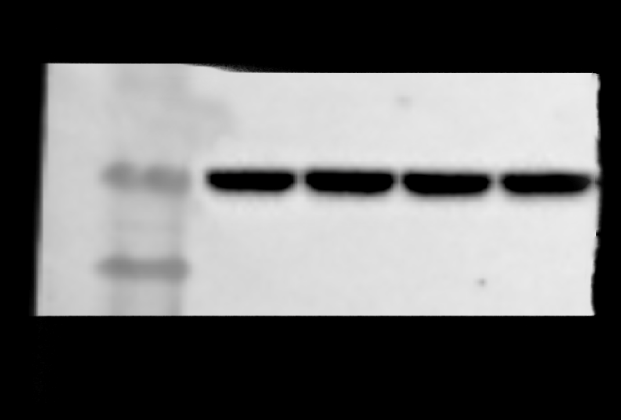


**IL-12p40**


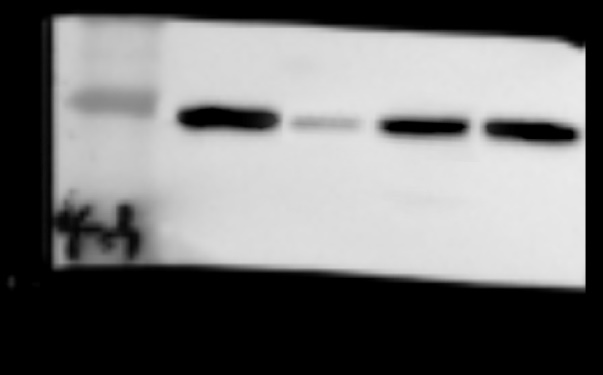


**GAPDH for IL-12 p40**


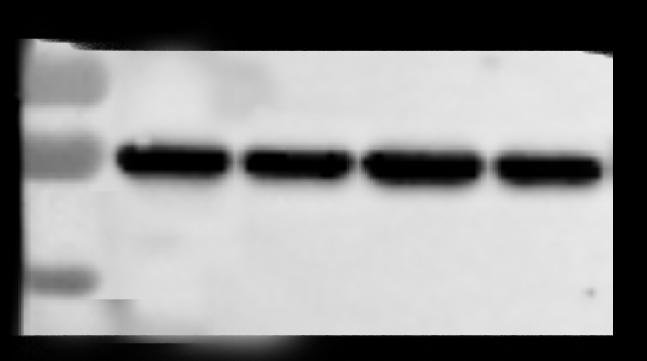


**Full gel pictures for Supplementary Figure 3**

**Ang1**


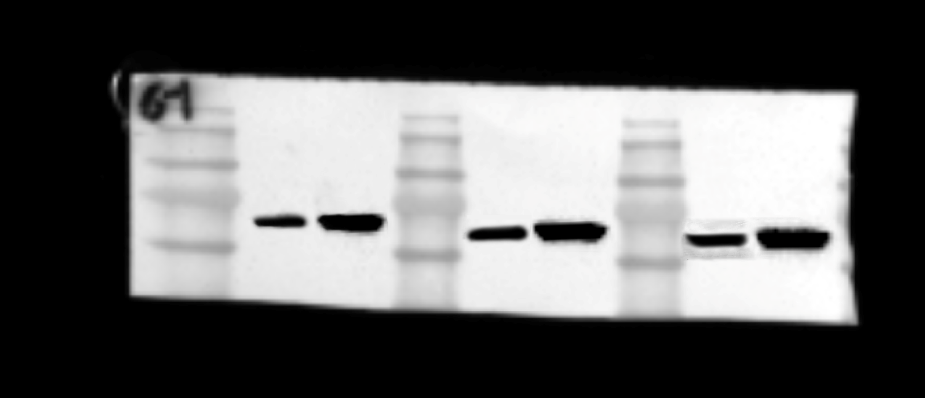


**GAPDH for Ang1**


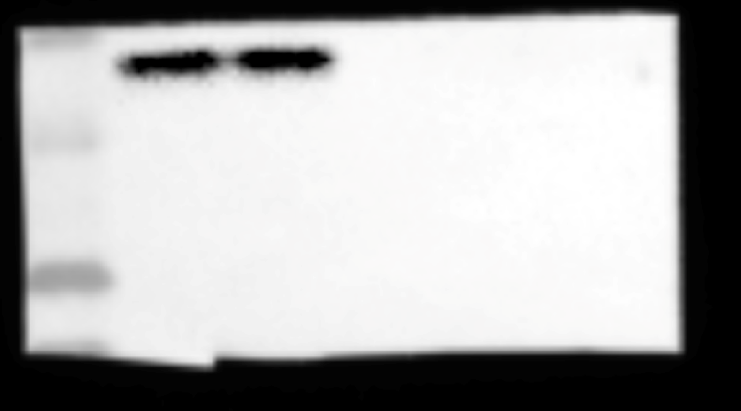


**p65**


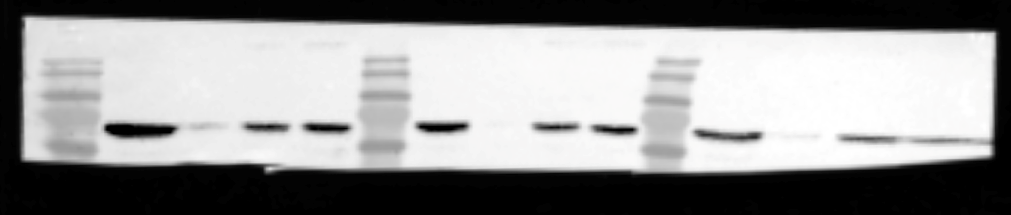


**GAPDH for p65**


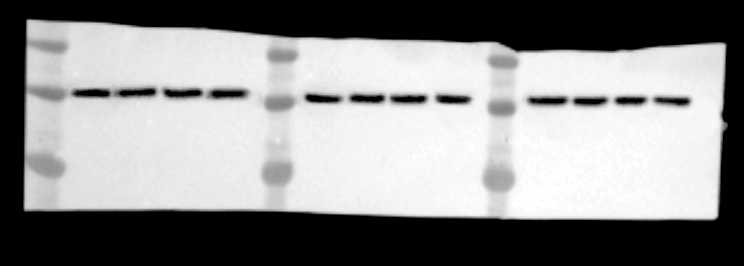

Supplement: Supplementary file 1 — Supplementary Information. [file 41598_2021_82531_MOESM1_ESM.docx]
